# Utility of CT assessment in hematology patients with invasive aspergillosis: a post-hoc analysis of phase 3 data

**DOI:** 10.1186/s12879-019-4039-7

**Published:** 2019-05-28

**Authors:** Jie Jin, Depei Wu, Yang Liu, Sisi Pan, Jean Li Yan, Jalal A. Aram, Yin-jun Lou, Haitao Meng, Xiaochen Chen, Xian’an Zhang, Ilan S. Schwartz, Thomas F. Patterson

**Affiliations:** 1 Department of Hematology, First Affiliated Hospital, Zhejiang University College of Medicine, Hangzhou, China; 2 Department of Hematology, First Affiliated Hospital, Soochow University, Suzhou, China; 3 Pfizer Inc, Beijing, China; 4 Pfizer Inc, New York, USA; 5 Division of Infectious Diseases, Department of Medicine, University of Alberta, Edmonton, Alberta Canada; 6 Division of Infectious Diseases, UT Health Science San Antonio, San Antonio, TX USA; 7 South Texas Veterans Health Care System, San Antonio, TX USA

**Keywords:** Invasive aspergillosis, Computed tomography scans, Diagnosis, Radiographic abnormalities, Antifungal treatment, Hematopoietic stem cell transplant, Hematologic malignancy

## Abstract

**Background:**

Pulmonary computed tomography (CT) scans are commonly used as part of the clinical criteria in diagnostic workup of invasive fungal diseases like invasive aspergillosis, and may identify radiographic abnormalities, such as halo signs or air-crescent signs. We assessed the diagnostic utility of CT assessment in patients with hematologic malignancies or those who had undergone allogeneic hematopoietic stem cell transplantation in whom invasive aspergillosis was suspected.

**Methods:**

This post-hoc analysis assessed data from a prospective, multicenter, international trial of voriconazole (with and without anidulafungin) in patients with suspected invasive aspergillosis (IA; proven, probable, or possible, using 2008 European Organisation for Research and Treatment of Cancer/Invasive Fungal Infections Cooperative Group and the National Institute of Allergy and Infectious Diseases Mycoses Study Group criteria) [NCT00531479]. Eligible patients received at least one baseline lung CT scan.

**Results:**

Of 395 patients included in this post-hoc analysis, 240 patients (60.8%) had ‘confirmed’ proven (9/240, 3.8%) or probable (231/240, 96.3%) invasive aspergillosis (cIA) and 155 patients (39.2%) had ‘non-confirmed’ invasive aspergillosis (all nIA; all possible IA (de Pauw et al., Clin Infect Dis 46:1813–21, 2008)). Mean age was 52.3 and 50.5 years, 56.3 and 60.0% of patients were male, and most patients were white (71.7 and 71.0%) in the cIA and nIA populations, respectively. Median baseline galactomannan was 1.4 (cIA) and 0.2 (nIA), mean Karnofsky score was 65.3 (cIA) and 66.8 (nIA), and mean baseline platelet count was 48.0 (cIA) and 314.1 (nIA). Pulmonary nodules (46.8% of all patients), bilateral lung lesions (37.5%), unilateral lung lesions (28.4%), and consolidation (24.8%) were the most common radiographic abnormalities. Ground-glass attenuation (cIA: 24.2%; nIA: 11.6%; *P* < 0.01) and pulmonary nodules (cIA: 52.5%; nIA: 38.1%; P < 0.01) were associated with cIA. Other chest CT scan abnormalities (including halo signs and air-crescent signs) at baseline in patients with hematologic malignancy or hematopoietic stem cell transplantation, and suspected IA, were not associated with cIA.

**Conclusions:**

These findings highlight the limitations in the sensitivity of chest CT scans for the diagnosis of IA, and reinforce the importance of incorporating other available clinical data to guide management decisions on individual patients, including whether empirical treatment is reasonable, pending full evaluation.

**Trial registration:**

NCT00531479 (First posted on ClinicalTrials.gov on September 18, 2007)

**Electronic supplementary material:**

The online version of this article (10.1186/s12879-019-4039-7) contains supplementary material, which is available to authorized users.

## Background

Invasive aspergillosis (IA) is an opportunistic infection caused by the inhalation of the ubiquitous and normally harmless *Aspergillus* conidia in immunocompromised individuals [1]. Despite advances in diagnosis and treatment, IA remains a life-threatening condition. One-year survival rates of just 25.4% have been reported for hematopoietic stem cell transplant (HSCT) recipients [2] and of 59% for organ transplant recipients [3].

The most recent guidance provided by the European Organisation for Research and Treatment of Cancer/Invasive Fungal Infections Cooperative Group and the National Institute of Allergy and Infectious Diseases Mycoses Study Group (EORTC/MSG) recommends that histopathologic, cytopathologic, or direct microscopic evidence be obtained to confirm a diagnosis of ‘proven’ invasive fungal disease [4]. However, given the rapid onset and serious threat of IA, empirical treatment is often used in neutropenic patients with persistent fever unresponsive to antibiotic therapy, prior to obtaining direct evidence of infection [5, 6].

Computed tomography (CT) scans of the chest are commonly used as part of the clinical criteria in diagnostic workup of invasive fungal diseases [4]. Radiographic signs on the CT scan, which have been documented to be associated with (although not completely specific for) pulmonary IA, include the so-called air-crescent sign and the halo sign [7]. Of note, the halo sign (a macronodule surrounded by a perimeter of ground-glass opacity) has been regarded as an early indicator of IA [8–10]. Furthermore, treatment based on the presence of the halo sign has been associated with earlier initiation of antifungal therapy, a better response to treatment, and improved survival [11, 12].

Although previous publications have highlighted the potential utility of CT-based diagnostics in IA, these have been used in cases with other clinical symptoms (for example, antibiotic-resistant neutropenic fever) [13] or in combination with microbiologic assessments, such as an enzyme immunoassay [6]. There are currently limited data regarding the utility of CT-based diagnosis in isolation to predict response to antifungal therapy. A study in China found that use of a high-resolution CT-driven antifungal strategy was effective and suitable for patients with hematologic malignancies [14]. Antifungal treatment was significantly more effective in patients with specific IA signs on pulmonary high-resolution CT in this study than for those with non-specific CT signs.

Here we report a post-hoc analysis of data from a phase 3 trial of patients with hematologic malignancies or who had undergone allogeneic HSCT, and suspected IA, to assess the diagnostic utility of radiographic signs at baseline obtained during chest CT scans in those who received antifungal treatment.

## Methods

### Study design

The objective of this post-hoc analysis was to determine the association between lesions and other findings on the baseline CT scan with the eventual diagnosis in patients with IA taking part in a phase 3 trial. The study, a prospective, double-blind, multicenter, international, randomized, placebo-controlled phase 3 trial, compared the efficacy and safety of treatment with voriconazole, with and without anidulafungin, in patients with suspected IA. The study was conducted in accordance with the International Conference on Harmonization Good Clinical Practice Guidelines and ethical principles originating in or derived from the Declaration of Helsinki, approved by the appropriate institutional review boards, and registered on ClinicalTrials.gov (NCT00531479). All patients provided written, informed consent. The results of the primary analysis, including patient eligibility criteria and dosing, have been previously reported [15].

Briefly, this study enrolled patients with hematologic malignancies or who had undergone allogeneic HSCT, and had suspected IA, which could be proven, probable, or possible, as defined by EORTC/MSG 2008 criteria [4]. All patients with possible IA were enrolled but were required to have a proven/probable diagnosis established within 7 days of enrolment. In total, 454 patients were randomly assigned and received at least 1 dose of treatment with voriconazole and anidulafungin (*n* = 228) or voriconazole monotherapy (*n* = 226) for up to 6 weeks. Voriconazole was administered intravenously on Week 1 (6 mg/kg of body weight every 12 h on the first day, then 4 mg/kg for the remainder of the week). Switching to oral voriconazole (300 mg every 12 h) was permitted, at the discretion of the investigator, for the remaining 5 weeks. In addition to voriconazole, all patients received either intravenous anidulafungin (200 mg on the first day, then 100 mg daily thereafter) or placebo for between 2 weeks (minimum) and 4 weeks (maximum). The primary analysis (all-cause mortality at Week 6) was carried out in the modified intention-to-treat population (mITT), which included 277 patients with confirmed proven or probable IA [15].

### Post-hoc analysis

The present post-hoc analysis included all patients in the mITT population with at least one CT scan of the lungs at baseline. CT scans carried out as standard of care within 5 days before enrolment could be used as the baseline scan. Modified EORTC/MSG criteria were used for diagnosis of proven, probable, or possible IA. Proven IA was defined as: histopathologic, cytopathologic, or direct microscopic examination of a needle aspiration or biopsy specimen showing hyphal forms with evidence of associated tissue damage (microscopically or as an infiltrate or lesion by imaging) or recovery of *Aspergillus* species from a normally sterile site. Probable IA required at least one host factor, one clinical criterion, and one microbiologic criterion. Patients with proven or probable IA were assigned ‘confirmed’ IA (cIA) and all others as ‘non-confirmed’ IA (nIA). This assessment was conducted by an independent review committee. For the purposes of the post-hoc analysis, patients were also grouped into those with cIA or nIA regardless of treatment arm.

Patient records, as provided by the investigators, were retrospectively reviewed to identify physician notes describing the presence of observable lesions or abnormalities on a CT scan at baseline. These were collated under common terms and the prevalence of each individual abnormality was assessed in both cIA and nIA groups.

### Endpoints

The following endpoints, as determined by a blinded, independent review committee, were assessed in cIA and nIA patient groups at Week 6: all-cause mortality (primary endpoint); clinical responses; radiographic responses; and global responses. Mortality rate was based on Kaplan-Meier product limit estimators, with statistical comparisons for the difference in mortality rates achieved using a one-sided *P* value. A sub-analysis comparing patients of Asian ethnicity with those of non-Asian ethnicity was also conducted. The sub-analysis was performed because an earlier post-hoc analysis suggested that race may be one covariate influencing global response to therapy [16]. This may be related to the high prevalence in Asian populations of a genetic polymorphism of the CYP2C19 enzyme that can reduce voriconazole metabolism [17–19].

Clinical response was defined as complete (sign/symptom resolution), partial (clinical improvement), stable (no discernible change), or failure (a worsening of signs/symptoms). A summary of clinical response at Week 6, which categorized successful responses as complete or partial, was determined by the investigator. Radiographic response at Week 6 was defined as successful (which was categorized as complete [> 90% radiographic improvement] or partial [50–90% radiographic improvement]); stable (< 50% radiographic improvement); or failure (worsening disease). Global responses were assessed by the investigator at Weeks 2, 4, 6, and end of treatment, and were categorized as one of four outcomes: complete (complete clinical and radiographic response), partial (clinical improvement and complete/partial radiographic response), stable (stable clinical or radiographic response), or failure (worsening disease). A summary of global responses at Week 6 was provided by the investigator. CT scan findings at baseline, and their potential utility and diagnostic significance for IA, were evaluated using Fisher’s exact test.

## Results

### Patients

A total of 395 patients were included in the post-hoc analysis; 240 patients (60.8%) were categorized as cIA, of which 9/240 (3.8%) had proven IA and 231/240 (96.3%) had probable IA. The remaining 155 patients (39.2%) were categorized as nIA, all of which were considered possible IA. The gender and ethnicity of patients were comparable between the cIA and nIA groups, with a slightly lower mean age observed for females categorized as nIA than for males or patients with cIA (Table 1).

**Table 1:** Baseline demographics and characteristics

	cIA			nIA		
	Male (*n* = 135)	Female (*n* = 105)	Total (*n* = 240)	Male (*n* = 93)	Female (*n* = 62)	Total (*n* = 155)
Mean age, years (SD)	52.1 (16.1)	52.6 (14.4)	52.3 (15.4)	53.5 (16.1)	46.2 (16.4)	50.5 (16.5)
Race, n (%)						
White	100 (74.1)	72 (68.6)	172 (71.7)	63 (67.7)	47 (75.8)	110 (71.0)
Black	0 (0.0)	2 (1.9)	2 (0.8)	1 (1.1)	0 (0.0)	1 (0.6)
Asian	34 (25.2)	28 (26.7)	62 (25.8)	26 (28.0)	13 (21.0)	39 (25.2)
Other	1 (0.7)	3 (2.9)	4 (1.7)	3 (3.2)	2 (3.2)	5 (3.2)
Mean weight, kg (SD)	73.4 (15.3)	62.1 (15.8)	68.5 (16.5)	75.3 (17.4)	63.4 (15.8)	70.5 (17.7)
Mean BMI, kg/m^2^ (SD)	23.9 (4.4)	24.2 (5.6)	24.0 (5.0)	24.7 (4.9)	24.3 (5.1)	24.5 (5.0)
Mean height, cm (SD)	174.8 (8.0)	160.1 (7.7)	168.3 (10.7)	173.9 (8.9)	160.9 (6.6)	168.6 (10.3)
Baseline serum/BAL GM, median (range)	–	–	1.4 (0.0–11.2)	–	–	0.2 (0.0–7.6)
Karnofsky score, mean (range)	–	–	65.3 (20.0–100.0)	–	–	66.8 (20.0–100.0)
Baseline platelet count, mean (range)	–	–	48.0 (1.0–305.0)	–	–	314.1 (2.0–34,000)

*BAL* Bronchoalveolar lavage, *BMI* Body mass index, *cIA* ‘Confirmed’ invasive aspergillosis, *GM* Galactomannan, *nIA* ‘Non-confirmed’ invasive aspergillosis, *SD* Standard deviation

### Radiographic signs and outcomes

**Fig. 1:**
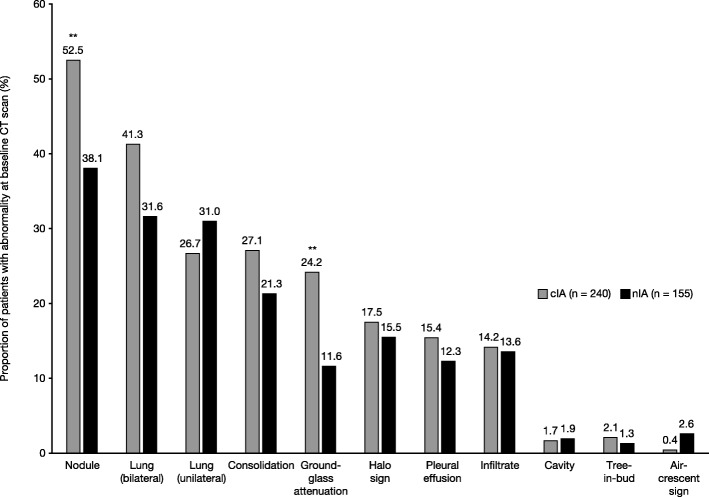
Radiographic abnormalities among patients with cIA and nIA. **P < 0.01 based on Fisher’s exact test. cIA. ‘Confirmed’ invasive aspergillosis, CT computed tomography, nIA ‘Non-confirmed’ invasive aspergillosis

The most commonly observed radiographic abnormalities seen across all patients in this post-hoc analysis were nodules (*n* = 185; 46.8%), bilateral lung lesions (*n* = 148; 37.5%), unilateral lung lesions (*n* = 112; 28.4%), and consolidation (*n* = 98; 24.8%) (Fig. 1). Other radiographic abnormalities observed were halo sign (*n* = 66; 16.7%), cavity (*n* = 7; 1.8%), and air-crescent sign (*n* = 5; 1.3%). Ground-glass attenuation was the radiographic abnormality most closely associated with subsequent confirmation of IA, observed more commonly in the cIA group (*n* = 58; 24.2%) than in the nIA group (*n* = 18; 11.6%) (*P* < 0.01). Pulmonary nodules were also observed more commonly in the cIA group (*n* = 126; 52.5%) than in the nIA group (*n* = 59; 38.1%) (*P* < 0.01). Although the presence of bilateral lung lesions was also more common in cIA (*n* = 99; 41.3%) than in nIA (*n* = 49; 31.6%) patients, statistical significance was not reached (*P* > 0.05). No other radiographic abnormalities appeared to be associated with the confirmation of IA.

### Mortality

**Fig. 2:**
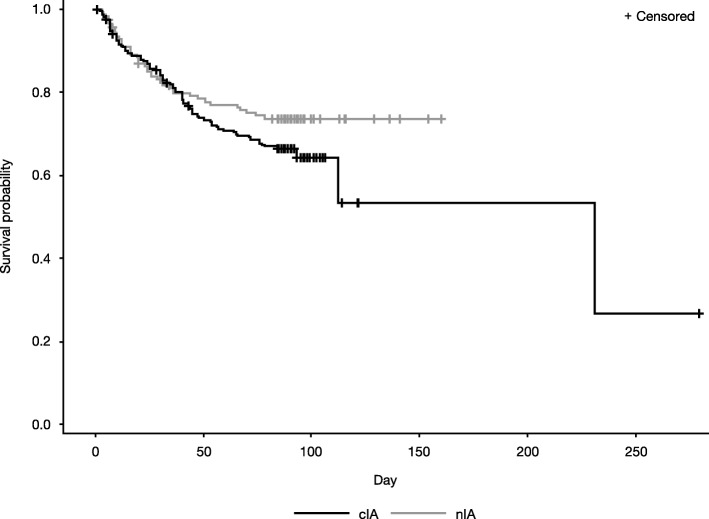
Kaplan-Meier plots for patients with cIA and nIA. cIA ‘Confirmed’ invasive aspergillosis, nIA ‘Non-confirmed’ invasive aspergillosis

**Fig. 3:**
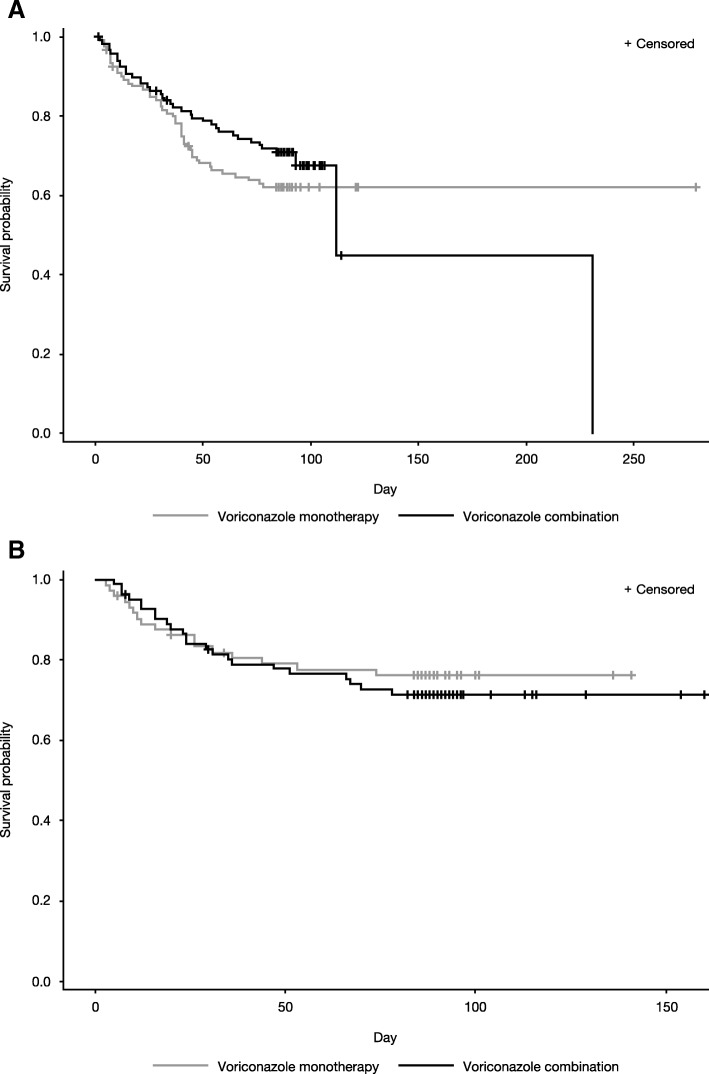
Kaplan-Meier plots for voriconazole monotherapy and combination therapy for patients with (A) cIA and (B) nIA. cIA ‘Confirmed’ invasive aspergillosis, nIA ‘Non-confirmed’ invasive aspergillosis

At Week 6, 85 patients in the mITT population had died (all-cause mortality). Mortality at 6 weeks was comparable between patients with cIA and nIA (22.8 vs 20.3% respectively [95% confidence interval for difference, − 5.83–10.84; *P* > 0.05]) (Table 2). After 6 weeks, survival probability for patients with cIA was lower than for patients with nIA (Fig. 2). Kaplan-Meier survival curves were generally similar between patients receiving monotherapy and combination therapy (Fig. 3).

**Table 2:** Mortality and clinical, radiographic, and global responses at Week 6

	cIA (*N* = 240)	nIA (*N* = 155)
Mortality, n (mortality rate,^a^ %)	54 (22.8)	31 (20.3)
Asian subpopulation	*n* = 60	*n* = 37
Mortality, n (mortality rate,^a^ %)	14 (23.9)	8 (22.2)
Non-Asian subpopulation	*n* = 180	*n* = 118
Mortality, n (mortality rate,^a^ %)	40 (22.5)	23 (19.8)
Clinical responses, n^b^ (%)	*n* = 177	*n* = 88
Complete (sign/symptom resolution)	105 (59.3)	42 (47.7)
Partial (clinical improvement)	39 (22.0)	18 (20.5)
Stable (no discernible change)	15 (8.5)	7 (8.0)
Failure (worsening of signs/symptoms)	7 (4.0)	4 (4.5)
Radiographic responses, n^c^ (%)	*n* = 177	*n* = 88
Complete (> 90% radiographic improvement)	35 (19.8)	22 (25.0)
Partial (50–90% radiographic improvement)	81 (45.8)	34 (38.6)
Stable (< 50% radiographic improvement)	37 (20.9)	10 (11.4)
Failure (worsening disease)	12 (6.8)	5 (5.7)
Global responses,^d^ n (%)	*n* = 240	*n* = 155
Complete	20 (8.3)	11 (7.1)
Partial	73 (30.4)	15 (9.7)
Stable	39 (16.3)	8 (5.2)
Failure	13 (5.4)	4 (2.6)

^a^Based on Kaplan-Meier product limit estimators. ^b^Missing data from 11 patients with cIA and 17 patients with nIA

^c^Missing data from 12 patients with cIA and 17 patients with nIA

^d^Global responses were categorized as complete (complete clinical and radiographic response), partial (clinical improvement and complete/partial radiographic response), stable (stable clinical or radiographic response), or failure (worsening disease); missing data from 95 patients with cIA and 117 patients with nIA.*cIA* ‘Confirmed’ invasive aspergillosis, *nIA* ‘Non-confirmed’ invasive aspergillosis

Among the Asian and non-Asian subpopulations, mortality at 6 weeks was comparable between patients with cIA and nIA (Asian subpopulation: 23.9 vs 22.2%; non-Asian subpopulation: 22.5 vs 19.8%, in cIA and nIA groups, respectively) (Table 2).

### Clinical, radiographic, and global responses

A complete or partial clinical response was observed at Week 6 in 81.4 and 68.2% of patients (with cIA and nIA, respectively) and a complete or partial radiographic response was achieved in 65.5 and 63.6% of patients (with cIA and nIA, respectively) (Table 2). More patients achieved a complete or partial global response with cIA (38.8%) than those with nIA (16.8%).

## Discussion

In this study, chest CT abnormalities of nodules and ground-glass attenuation observed at baseline in patients with hematologic malignancy or allogeneic HSCT, and suspected IA, were associated with subsequent confirmation of IA. In contrast, other radiographic findings (including halo and air-crescent signs) at baseline were not associated with subsequent confirmation of IA in these patients. This finding is in contrast to a previous study that showed abnormalities such as halo and air-crescent signs to be associated with IA diagnosis [11].

Nevertheless, these findings do further support the applicability of radiographic abnormalities as a clinical tool for the diagnosis of IA. The use of specific radiographic abnormalities to guide decisions on pre-emptive therapy previously appeared to reduce overtreatment of patients with possible IA. For example, a reduced rate of antifungal use from 35 to 7.7% was observed when chest CT scans were included as part of a pre-emptive therapy approach in neutropenic patients at risk of invasive fungal infection [6]. Notably, of the patients who received pre-emptive therapy, the majority exhibited halo signs at the first CT scan (*n*/*N* = 13/19; 68.4%) and about half showed air-crescent signs in a subsequent CT scan (*n*/*N* = 9/19; 47.4%). Other CT abnormalities at baseline or follow-up scan in patients receiving pre-emptive therapy included nodular lesions, consolidation, pneumothorax, and cavern, but these were less frequent (*n/N* = 1/19; 5.3% each) [6].

It is worth noting that proven or probable invasive fungal infection appears to be more common in those patients receiving pre-emptive therapy, compared with those receiving empirical therapy [5], further highlighting the benefit of refining pre-emptive therapy approaches for patients with IA. The use of CT scans as clinical tools in IA diagnosis would potentially have implications for current clinical guidelines (EORTC/MSG, Infectious Diseases Society of America, European Conference on Infections in Leukemia) [4, 20, 21]. It is also worth considering that complementary CT pulmonary angiography may improve the sensitivity of CT scan-based IA diagnoses in patients with hematologic malignancy [22].

In the analysis described here, there were no statistically significant differences between the cIA and nIA groups in terms of mortality at 6 weeks and no apparent differences in radiographic progression at 6 weeks. Mortality rates in Asian and non-Asian subpopulations were similar to overall mortality, in both cIA and nIA groups. In the cIA group, however, the proportion of patients achieving complete clinical and global responses were numerically greater than in the nIA group.

To the authors’ knowledge, this analysis is the largest assessment of radiographic progression in patients receiving antifungal therapy for the treatment of IA. Blinded assessment of the infection, and of the clinical and radiologic outcomes, was performed by a committee of experts, which minimized the potential for bias.

This study had some important limitations that should be considered. The proportions of patients in this analysis who had radiographic signs were lower than those previously reported, particularly for halo signs. In this study, 16.7% of patients presented with halo signs, compared with between 60.9 and ~ 100.0% of patients in previous studies [12, 23]. One possible explanation for this discrepancy is the method of radiographic interpretation. In order to make the findings of this post-hoc analysis of a clinical trial generalizable to real-world clinical scenarios, and considering that treatment decisions for immunocompromised patients with suspected IA do not necessarily await radiologist interpretation, we classified radiographic findings according to documentation in clinical charts, which were not necessarily the observations of radiologists. In contrast, other studies reporting higher rates of radiographic lesions have relied on the interpretation of radiologists [12]. Moreover, although the risk of misclassification of radiographic signs may not have been systematically different between patients classified as cIA and nIA, nor between treatment arms, if non-radiologists are less (or more) likely to document the presence of certain signs, the power of this study to observe differences between patients who were later classified as cIA or nIA may have been affected. It is also worth noting that patients with possible IA and radiographic abnormalities may have had IA infection, even if further investigation failed to upgrade the diagnosis to proven/probable IA; this may have resulted in an underestimation of the discriminative value of chest CT scans. Overall, the results should be interpreted with caution as this is a post-hoc analysis and further confirmative studies are warranted.

## Conclusions

In conclusion, it appears that once a patient meets minimal EORTC/MSG criteria for possible IA, differentiating between those with or without confirmed IA based on baseline CT scan can be difficult. It was interesting to observe that radiographic abnormalities from chest CT scans, such as halo and air-crescent signs traditionally associated with IA, were generally not associated with subsequent confirmation of IA. However, this could be due to the low proportion of patients presenting with such signs, so caution should be applied when interpreting the diagnostic utility of these abnormalities in the context of this study. Abnormalities observed in this analysis associated with cIA include ground-glass attenuation and nodules as well as, to a lesser degree, bilateral lesions. Taken together with findings from other groups [6, 13, 14, 24], this suggests that radiographic abnormalities should be interpreted in conjunction with other available clinical data to guide management decisions on individual patients, including whether treatment is reasonable, pending full evaluation. Considering the high mortality rate associated with IA, patients may need to be managed as having IA until infection has been ruled out.

## Additional file

Additional file 1: List of ethics committees or institutional review boards of the investigational centers. This file provides a list of the investigational center numbers and their associated Institutional Review Boards or Independent Ethics Committees. (DOCX 48 kb)

## Abbreviations

BMI: Body mass index
cIA: Confirmed invasive aspergillosis
CT: Computed tomography
CYP: Cytochrome P450
EORTC: European Organisation for Research and Treatment of Cancer
HSCT: Hematopoietic stem cell transplantation
IA: Invasive aspergillosis
mITT: Modified intent-to-treat
MSG: (National Institute of Allergy and Infectious Diseases) Mycoses Study Group
nIA: Non-confirmed invasive aspergillosis
SD: Standard deviation
