# Supplementary material for: Utility of CT assessment in hematology patients with invasive aspergillosis: a post-hoc analysis of phase 3 data
Source: BMC Infect Dis. 2019 May 28;19:471. doi: 10.1186/s12879-019-4039-7 (PMC6537389; doi:10.1186/s12879-019-4039-7)
Supplement: Supplementary file 1 — List of ethics committees or institutional review boards of the investigational centers. This file provides a list of the investigational center numbers and their associated Institutional Review Boards or Independent Ethics Committees. (DOCX 48 kb) [file 12879_2019_4039_MOESM1_ESM.docx]

# LIST OF ETHICS COMMITTEES OR INSTITUTIONAL REVIEW BOARDS OF THE INVESTIGATIONAL CENTERS

## Australia

| **Center** | **Institutional Review Board or Ethics Committee Address(es)** |
| --- | --- |
| 1089 | Sydney West Area Health Service (SWAHS) Human Research Ethics Committee - Westmead Campus  Research Office Westmead Hospital  Cnr Hawkesbury Road and Darcy Roads  Westmead, NSW 2415  AUSTRALIA |
| 1090 | The Royal Brisbane and Women's Hospital Health Service District Human Research Ethics Committee  Royal Brisbane and Women's Hospital  Herston Road  Herston, QLD 4029  AUSTRALIA |
| 1091 * | Royal Adelaide Hospital Research Ethics Committee  Royal Adelaide Hospital  Level 3, Hanson Centre  North Terrace  Adelaide, SA 5000  AUSTRALIA |
| 1187 * | The Alfred Hospital Ethics Committee  Alfred Hospital  Commercial Road  Melbourne, VIC 3004  AUSTRALIA |

## Austria

| **Center** | **Institutional Review Board or Ethics Committee Address(es)** |
| --- | --- |
| 1035 * | Ethikkommission der Medizinischen Universitaet Wien & d. Allgemeinen Krankenhauses d. Stadt Wien-AKH  Borschkegasse 8b/E 06  Wien, A-1090  AUSTRIA |
| 1036 * | Ethikkommission der Medizinischen Universitaet Wien & d. Allgemeinen Krankenhauses d. Stadt Wien-AKH  Borschkegasse 8b/E 06  Wien, A-1090  AUSTRIA |

## Belgium

| **Center** | **Institutional Review Board or Ethics Committee Address(es)** |
| --- | --- |
| 1130 | Cliniques Universitaires Saint-Luc  Commission d'Etique Biomédicale Hospitalo-Facultaire  Avenue Hippocrate 55.14  Tour Harvey - niveau 0  Bruxelles, 1200  BELGIUM |
| 1131 * | Cliniques Universitaires Saint-Luc  Commission d'Etique Biomédicale Hospitalo-Facultaire  Avenue Hippocrate 55.14  Tour Harvey - niveau 0  Bruxelles, 1200  BELGIUM |
| 1132 | Cliniques Universitaires Saint-Luc  Commission d'Etique Biomédicale Hospitalo-Facultaire  Avenue Hippocrate 55.14  Tour Harvey - niveau 0  Bruxelles, 1200  BELGIUM |
| 1133 * | Cliniques Universitaires Saint-Luc  Commission d'Etique Biomédicale Hospitalo-Facultaire  Avenue Hippocrate 55.14  Tour Harvey - niveau 0  Bruxelles, 1200  BELGIUM |
| 1134 | Cliniques Universitaires Saint-Luc  Commission d'Etique Biomédicale Hospitalo-Facultaire  Avenue Hippocrate 55.14  Tour Harvey - niveau 0  Bruxelles, 1200  BELGIUM |
| 1167 * | Cliniques Universitaires Saint-Luc  Commission d'Etique Biomédicale Hospitalo-Facultaire  Avenue Hippocrate 55.14  Tour Harvey - niveau 0  Bruxelles, 1200  BELGIUM |
| 1177 | Cliniques Universitaires Saint-Luc  Commission d'Etique Biomédicale Hospitalo-Facultaire  Avenue Hippocrate 55.14  Tour Harvey - niveau 0  Bruxelles, 1200  BELGIUM |

## Brazil

| **Center** | **Institutional Review Board or Ethics Committee Address(es)** |
| --- | --- |
| 1092 | Comite de Etica em Pesquisa em Seres Humanos do HC-UFPR  Rua General Carneiro, 181  Curitiba, PR 80060-900  BRAZIL |
| 1103 | Comite de Etica em Pesquisa do Hospital Universitario Clementino Fraga Filho - UFRJ  Rua Professor Rodolpho Paulo Rocco, 255  1 andar, sl. 1D-46 - Cidade Universitaria  Rio de Janeiro, RJ 21941-617  BRAZIL |
| 1127 * | Comite de Etica em Pesquisa do Instituto Nacional de Cancer - INCA  Rua André Cavalcanti, 37 - 2º andar - Centro  Rio de Janeiro, RJ 20231-050  BRAZIL |
| 1128 * | Comite de Etica em Pesquisa do Hospital Alemao Oswaldo Cruz  Rua Joao Juliao, 331 - 14º andar  Bela Vista  Sao Paulo, SP 01323-903  BRAZIL |
| 1171 * | Comite de Etica em Pesquisa da Universidade Federal de Sao Paulo/ Hospital Sao Paulo  Rua Botucatu  572, 1 andar , conjunto 14 - Vila Clementino  São Paulo, SP 04023-062  BRAZIL |
| 1172 * | Comite de Etica em Pesquisa do Hospital Israelita Albert Einstein  Avenida Albert Einstein, 627 / 701 - Bloco A - 2o Subsolo  Morumbi  São Paulo, São Paulo 05651-901  BRAZIL |
| 1185 | Comite de Etica em Pesquisa em Seres Humanos  Comite de Etica da Irmandade da Santa Casa de Misericordia de Porto Alegre  Rua Professor Annes Dias, 285  Porto Alegre, RS 90020-090  BRAZIL |

## Canada

| **Center** | **Institutional Review Board or Ethics Committee Address(es)** |
| --- | --- |
| 1001 | University of Manitoba  Biomedical Research Ethics Board  P126 Pathology Building  770 Bannatyne Avenue  Winnipeg, MB R3E 0W3  CANADA |
| 1004 | Comite D'Ethique de la Recherche de l'Hopital Maisonneuve-Rosemont  5415 Boulevard L'Assomption  Montreal, QC H1T 2M4  CANADA |
| 1006 | Hamilton Health Sciences  Research Ethics Board  Suite 102  293 Wellington  Hamilton, ON L8L 8E7  CANADA |
| 1007 | Research Ethics Board, Hopital St-Francois D'Assise  Research Ethics Board Counsellor, Marie Claude Bernier  Local A0-124  10 rue de l'Espinay  Quebec, QC G1L 3L5  CANADA |
| 1030 * | Health Research Ethics Board  University of Alberta  213 Heritage Medical Research Centre  Edmonton, AB T6G 2S2  CANADA |
| 1031 * | Comite de la Recherche et Comite d'Ethique de la Recherche  Edifice Cooper  Bureau M-207  3981 St-Laurent, Mezzanine 2  Montreal, QC H2W 1Y5  CANADA |
| 1032 * | The Ottawa Hospital Research Ethics Board  Suite 106  751 Parkdale Avenue  Ottawa, ON K1Y 1J7  CANADA |

## Czech Republic

| **Center** | **Institutional Review Board or Ethics Committee Address(es)** |
| --- | --- |
| 1152 | Eticka komise pro multicentricka klinicka hodnoceni Fakultni nemocnice v Motole  V Uvalu 84  150 06  Praha 5,  CZECH REPUBLIC |
|  | Eticka komise UHKT  U nemocnice 1  Praha 2, 128 20  CZECH REPUBLIC |

## France

| **Center** | **Institutional Review Board or Ethics Committee Address(es)** |
| --- | --- |
| 1107 | CPP Est IV  1 place de l'Hôpital  Strasbourg cedex, 67091  FRANCE |
| 1108 | CPP Est IV  1 place de l'Hôpital  Strasbourg cedex, 67091  FRANCE |
| 1109 | CPP Est IV  1 place de l'Hôpital  Strasbourg cedex, 67091  FRANCE |
| 1110 * | CPP Est IV  1 place de l'Hôpital  Strasbourg cedex, 67091  FRANCE |
| 1111 | CPP Est IV  1 place de l'Hôpital  Strasbourg cedex, 67091  FRANCE |
| 1112 * | CPP Est IV  1 place de l'Hôpital  Strasbourg cedex, 67091  FRANCE |
| 1122 | CPP Est IV  1 place de l'Hôpital  Strasbourg cedex, 67091  FRANCE |
| 1123 * | CPP Est IV  1 place de l'Hôpital  Strasbourg cedex, 67091  FRANCE |
| 1139 * | CPP EST IV  Hôpital civil  Bâtiment de la Direction Générale  1 place de l'hôpital  BP 426  STRASBOURG, 67091  FRANCE |
| 1148 | CPP Est IV  1 place de l'Hôpital  Strasbourg cedex, 67091  FRANCE |
| 1166 * | CPP Est IV  1 place de l'Hôpital  Strasbourg cedex, 67091  FRANCE |
| 1168 | CPP Est IV  1 place de l'Hôpital  Strasbourg cedex, 67091  FRANCE |
| 1192 | CPP Est IV  1 place de l'Hôpital  Strasbourg cedex, 67091  FRANCE |

## Germany

| **Center** | **Institutional Review Board or Ethics Committee Address(es)** |
| --- | --- |
| 1022 | Ethik-Kommission der Medizinischen Fakultaet der Ludwig-Maximilians Universitaet Muenchen  Pettenkoferstr. 8  Muenchen, 80336  GERMANY |
| 1023 | Ethik-Kommission der Medizinischen Fakultaet der Ludwig-Maximilians Universitaet Muenchen  Pettenkoferstr. 8  Muenchen, 80336  GERMANY |
| 1024 * | Ethik-Kommission der Medizinischen Fakultaet der Ludwig-Maximilians Universitaet Muenchen  Pettenkoferstr. 8  Muenchen, 80336  GERMANY |
| 1025 | Ethik-Kommission der Medizinischen Fakultaet der Ludwig-Maximilians Universitaet Muenchen  Pettenkoferstr. 8  Muenchen, 80336  GERMANY |
| 1026 | Ethik-Kommission der Medizinischen Fakultaet der Ludwig-Maximilians Universitaet Muenchen  Pettenkoferstr. 8  Muenchen, 80336  GERMANY |
| 1037 * | Ethik-Kommission der Medizinischen Fakultaet der Ludwig-Maximilians Universitaet Muenchen  Marchioninistrasse 15  Muenchen, 81377  GERMANY |
| 1042 | Ethik-Kommission der Medizinischen Fakultaet der Ludwig-Maximilians Universitaet Muenchen  Pettenkoferstr. 8  Muenchen, 80336  GERMANY |
| 1043 | Ethik-Kommission der Medizinischen Fakultaet der Ludwig-Maximilians Universitaet Muenchen  Pettenkoferstr. 8  Muenchen, 80336  GERMANY |
| 1044 * | Ethik-Kommission der Medizinischen Fakultaet der Ludwig-Maximilians Universitaet Muenchen  Pettenkoferstr. 8  Muenchen, 80336  GERMANY |
| 1057 * | Ethik-Kommission der Medizinischen Fakultaet der Ludwig-Maximilians Universitaet Muenchen  Pettenkoferstr. 8  Muenchen, 80336  GERMANY |
| 1058 * | Ethik-Kommission der Medizinischen Fakultaet der Ludwig-Maximilians Universitaet Muenchen  Marchioninistrasse 15  Muenchen, 81377  GERMANY |
| 1071 | Ethik-Kommission der Medizinischen Fakultaet der Ludwig-Maximilians Universitaet Muenchen  Pettenkoferstr. 8  Muenchen, 80336  GERMANY |
| 1080 * | Ethik-Kommission der Medizinischen Fakultaet der Ludwig-Maximilians Universitaet Muenchen  Marchioninistrasse 15  Muenchen, 81377  GERMANY |
| 1081 * | Ethik-Kommission der Medizinischen Fakultaet der Ludwig-Maximilians Universitaet Muenchen  Marchioninistrasse 15  Muenchen, 81377  GERMANY |
| 1082 | Ethik-Kommission der Medizinischen Fakultaet der Ludwig-Maximilians Universitaet Muenchen  Pettenkoferstr. 8  Muenchen, 80336  GERMANY |
| 1087 | Ethik-Kommission der Medizinischen Fakultaet der Ludwig-Maximilians Universitaet Muenchen  Pettenkoferstr. 8  Muenchen, 80336  GERMANY |
| 1113 | Ethik-Kommission der Medizinischen Fakultaet der Ludwig-Maximilians Universitaet Muenchen  Pettenkoferstr. 8  Muenchen, 80336  GERMANY |
| 1182 * | Ethik-Kommission der Medizinischen Fakultaet der Ludwig-Maximilians Universitaet Muenchen  Marchioninistrasse 15  Muenchen, 81377  GERMANY |
| 1184 | Ethik-Kommission der Medizinischen Fakultaet der Ludwig-Maximilians Universitaet Muenchen  Pettenkoferstr. 8  Muenchen, 80336  GERMANY |
| 1186 | Ethik-Kommission der Medizinischen Fakultaet der Ludwig-Maximilians Universitaet Muenchen  Pettenkoferstr. 8  Muenchen, 80336  GERMANY |
| 1209 * | Ethik-Kommission der Medizinischen Fakultaet der Ludwig-Maximilians Universitaet Muenchen  Marchioninistrasse 15  Muenchen, 81377  GERMANY |
| 1240 * | Ethik-Kommission der Medizinischen Fakultaet der Ludwig-Maximilians Universitaet Muenchen  Pettenkoferstr. 8  Muenchen, 80336  GERMANY |

## Greece

| **Center** | **Institutional Review Board or Ethics Committee Address(es)** |
| --- | --- |
| 1142 * | National Ethics Committee  284 Mesogion Avenue  Athens, 15562  GREECE |
| 1146 * | National Ethics Committee  284 Mesogion Avenue  Athens, 15562  GREECE |
| 1147 | National Ethics Committee  284 Mesogion Avenue  Athens, 15562  GREECE |

## India

| **Center** | **Institutional Review Board or Ethics Committee Address(es)** |
| --- | --- |
| 1226 | Sahyadri Speciality Hospitals Ethics Committee  Plot No. 30C  Erandawane, Karve Road  Pune,, Maharashtra 411004  INDIA |

## Italy

| **Center** | **Institutional Review Board or Ethics Committee Address(es)** |
| --- | --- |
| 1093 | Comitato Etico dell'Universita' Cattolica del S. Cuore, Policlinico Gemelli, Roma, Lazio  Largo Gemelli, 8  Roma, 00168  ITALY |
| 1094 | Comitato Etico per la sperimentazione clinica dei farmaci dell'ASL di Pescara, Abruzzo  Via Fonte Romana, 8  Pescara, 65100  ITALY |
| 1095 | Comitato Etico Interaziendale della ASO Santi Croce e Carle di Cuneo  e delle ASL 15 di Cuneo, 16 di Mondovi', 17 di Savigliano e 18 di Alba  Via Monte Zovetto, 18  Cuneo, 12100  ITALY |
| 1096 | Comitato Etico  Comitato Etico Scientifico dell'Azienda Ospedaliera Ospedale Niguarda Ca' Granda di Milano  Piazza Ospedale Maggiore, 3  Milano, 20162  ITALY |
| 1097 | Comitato Etico Azienda Ospedaliera Universitaria San Martino  Largo Rosanna Benzi 10  Genova, 16132  ITALY |
| 1104 | Comitato Etico delle Aziende Sanitarie Dell'Umbria  Via della Rivoluzione, 16  Ellera di Corciano (PG), 06070  ITALY |
| 1136 | Comitato Etico dell'IRCCS Fondazione San Raffaele del Monte Tabor di Milano  Via Olgettina, 60  Milano, 20132  ITALY |

## Korea, Republic Of

| **Center** | **Institutional Review Board or Ethics Committee Address(es)** |
| --- | --- |
| 1116 | IRB of Seoul National University Hospital  28 Yongon-dong Chongno-gu  Seoul, 110-744  KOREA, REPUBLIC OF |
| 1117 | IRB of Severance Hospital  Yonsei University College of Medicine, Severance hospital  250 Seongsanno, Seodaemun-Gu  Seoul, 120-752  KOREA, REPUBLIC OF |
| 1118 | Samsung Medical Center IRB  50 Irwon-dong, Gangnam-Gu  Seoul, 135-710  KOREA, REPUBLIC OF |
| 1119 | IRB of Asan Medical Center  388-1, Pungnap-2dong, Songpa-gu  Seoul, 138-736  KOREA, REPUBLIC OF |
| 1120 | IRB of The Catholic University of Korea Seoul St. Mary's Hospital  505 Banpo-dong, Seocho-gu  Seoul, 137-701  KOREA, REPUBLIC OF |

## Netherlands

| **Center** | **Institutional Review Board or Ethics Committee Address(es)** |
| --- | --- |
| 1138 | Academisch Ziekenhuis Leiden  Commissie Medische Ethiek  Albinusdreef 2  Leiden, 2333 ZA  NETHERLANDS |
| 1163 * | Academisch Ziekenhuis Leiden  Commissie Medische Ethiek  Albinusdreef 2  Leiden, 2333 ZA  NETHERLANDS |
| 1175 * | Academisch Ziekenhuis Leiden  Commissie Medische Ethiek  Albinusdreef 2  Leiden, 2333 ZA  NETHERLANDS |
| 1178 * | Academisch Ziekenhuis Leiden  Commissie Medische Ethiek  Albinusdreef 2  Leiden, 2333 ZA  NETHERLANDS |
| 1213 * | Academisch Ziekenhuis Leiden  Commissie Medische Ethiek  Albinusdreef 2  Leiden, 2333 ZA  NETHERLANDS |

## Peru

| **Center** | **Institutional Review Board or Ethics Committee Address(es)** |
| --- | --- |
| 1066 | Comite Institucional de Etica en Investigacion  Instituto Nacional de Enfermedades Neoplasicas  Avenida Angamos Este # 2520  Surquillo, Lima Lima 34  PRU |

## Poland

| **Center** | **Institutional Review Board or Ethics Committee Address(es)** |
| --- | --- |
| 1045 | Komisja Bioetyczna przy Warszawskim Uniwersytecie Medycznym w Warszawie  ul. Zwirki i Wigury 61  Warszawa, 02-091  POLAND |
| 1046 * | Komisja Bioetyczna przy Warszawskim Uniwersytecie Medycznym w Warszawie  ul. Zwirki i Wigury 61  Warszawa, 02-091  POLAND |
| 1048 * | Komisja Bioetyczna przy Warszawskim Uniwersytecie Medycznym w Warszawie  ul. Zwirki i Wigury 61  Warszawa, 02-091  POLAND |
| 1049 * | Komisja Bioetyczna przy Warszawskim Uniwersytecie Medycznym w Warszawie  ul. Zwirki i Wigury 61  Warszawa, 02-091  POLAND |
| 1050 | Komisja Bioetyczna przy Warszawskim Uniwersytecie Medycznym w Warszawie  ul. Zwirki i Wigury 61  Warszawa, 02-091  POLAND |
| 1052 * | Komisja Bioetyczna przy Warszawskim Uniwersytecie Medycznym w Warszawie  ul. Zwirki i Wigury 61  Warszawa, 02-091  POLAND |

## Portugal

| **Center** | **Institutional Review Board or Ethics Committee Address(es)** |
| --- | --- |
| 1143 | CEIC - Comissão de Ética para a Investigação Clínica  Parque da Saúde de Lisboa - Avª do Brasil, 53- Pavilhão 17-A  Lisboa, 1749-004  PORTUGAL |
| 1144 | CEIC - Comissão de Ética para a Investigação Clínica  Parque da Saúde de Lisboa - Avª do Brasil, 53- Pavilhão 17-A  Lisboa, 1749-004  PORTUGAL |
| 1216 * | CEIC - Comissão de Ética para a Investigação Clínica  Parque da Saúde de Lisboa - Avª do Brasil, 53- Pavilhão 17-A  Lisboa, 1749-004  PORTUGAL |
| 1217 * | CEIC - Comissão de Ética para a Investigação Clínica  Parque da Saúde de Lisboa - Avª do Brasil, 53- Pavilhão 17-A  Lisboa, 1749-004  PORTUGAL |

## Russian Federation

| **Center** | **Institutional Review Board or Ethics Committee Address(es)** |
| --- | --- |
| 1232 | Ethics Committee of RUSSIAN ONCOLOGICAL RESEARCH CENTER n.a. N.N. BLOKHIN RAMS  Kashirskoe shosse, 24  Moscow, 115478  RUSSIAN FEDERATION |
|  | Ethics Council at the Ministry of Healthcare and Social Development of Russian Federation  3, Rakhmanovskij per.  Moscow, 127994  RUSSIAN FEDERATION |
| 1233 | Ethics Committee of State Institution "Scientific center for Hematology RAMS"  4a, Novy Zykovskiy proezd  Moscow, 125167  RUSSIAN FEDERATION |
|  | Ethics Council at the Ministry of Healthcare and Social Development of Russian Federation  3, Rakhmanovskij per.  Moscow, 127994  RUSSIAN FEDERATION |
| 1234 * | Ethics Comittee of SPb MAPGE  41, Kirochnaya str  Saint-Petersburg, 191015  RUSSIAN FEDERATION |
|  | Ethics Committee at the Federal Service on Surveillance in Healthcare and Social Development  8, str. 2, Petrovskij bulvar  Moscow, 127051  RUSSIAN FEDERATION |
| 1235 | Ethic Committee at Fed. State Enterprise"Russian Defense Ministry Burdenko Main Military Hospital"  3, Hospital sq.  Moscow, 105229  RUSSIAN FEDERATION |
|  | Ethics Council at the Ministry of Healthcare and Social Development of Russian Federation  3, Rakhmanovskij per.  Moscow, 127994  RUSSIAN FEDERATION |
| 1236 | Ethics Council at the Ministry of Healthcare and Social Development of Russian Federation  3, Rakhmanovskij per.  Moscow, 127994  RUSSIAN FEDERATION |
|  | Local Ethics Committee at St. Petersburg Pavlov Medical University  Rentgena str. 10  St. Petersburg, 197101  RUSSIAN FEDERATION |
| 1241 * | Ethics Committee at the Federal Service on Surveillance in Healthcare and Social Development  8, str. 2, Petrovskij bulvar  Moscow, 127051  RUSSIAN FEDERATION |
|  | Local Ethics Committee of the City Oncology Clinical Hospital #62  P/o Stepanovskoe  Krasnogorskiy District, Moscow Region 143423  RUSSIAN FEDERATION |

## Saudi Arabia

| **Center** | **Institutional Review Board or Ethics Committee Address(es)** |
| --- | --- |
| 1214 * | King Faisal Specialist Hospital and Research Centre  Ethics Committee  Riyadh,  SAUDI ARABIA |

## Singapore

| **Center** | **Institutional Review Board or Ethics Committee Address(es)** |
| --- | --- |
| 1073 | SingHealth Centralized Institutional Review Board  Singapore Health Services Pte Ltd  Blk A, 7 Hospital Drive,  SingHealth Research Facilities, #03-01  Singapore, Singapore 169611  SINGAPORE |
| 1079 | Domain Specific Review Board (DSRB)  National Healthcare Group  Research & Development Office  6 Commonwealth Lane, Level 6 GMTI Building  Singapore, Singapore 149547  SINGAPORE |

## Spain

| **Center** | **Institutional Review Board or Ethics Committee Address(es)** |
| --- | --- |
| 1019 * | Hospital Universitario de la Princesa  ETHICS COMMITTEE OF CLINIC INVESTIGATION  C/ DIEGO DE LEON, 62  MADRID, MADRID 28006  SPAIN |
| 1060 | Hospital Universitario de la Princesa  ETHICS COMMITTEE OF CLINIC INVESTIGATION  C/ DIEGO DE LEON, 62  MADRID, MADRID 28006  SPAIN |
| 1061 | Hospital Universitario de la Princesa  ETHICS COMMITTEE OF CLINIC INVESTIGATION  C/ DIEGO DE LEON, 62  MADRID, MADRID 28006  SPAIN |
| 1062 | Hospital Universitario de la Princesa  ETHICS COMMITTEE OF CLINIC INVESTIGATION  C/ DIEGO DE LEON, 62  MADRID, MADRID 28006  SPAIN |
| 1063 | Hospital Universitario de la Princesa  ETHICS COMMITTEE OF CLINIC INVESTIGATION  C/ DIEGO DE LEON, 62  MADRID, MADRID 28006  SPAIN |
| 1072 * | Hospital Universitario de la Princesa  ETHICS COMMITTEE OF CLINIC INVESTIGATION  C/ DIEGO DE LEON, 62  MADRID, MADRID 28006  SPAIN |
| 1137 * | Hospital Universitario de la Princesa  ETHICS COMMITTEE OF CLINIC INVESTIGATION  C/ DIEGO DE LEON, 62  MADRID, MADRID 28006  SPAIN |
| 1181 * | Hospital Universitario de la Princesa  ETHICS COMMITTEE OF CLINIC INVESTIGATION  C/ DIEGO DE LEON, 62  MADRID, MADRID 28006  SPAIN |
| 1222 * | Hospital Universitario de la Princesa  ETHICS COMMITTEE OF CLINIC INVESTIGATION  C/ DIEGO DE LEON, 62  MADRID, MADRID 28006  SPAIN |
| 1223 | Hospital Universitario de la Princesa  ETHICS COMMITTEE OF CLINIC INVESTIGATION  C/ DIEGO DE LEON, 62  MADRID, MADRID 28006  SPAIN |
| 1224 * | Hospital Universitario de la Princesa  ETHICS COMMITTEE OF CLINIC INVESTIGATION  C/ DIEGO DE LEON, 62  MADRID, MADRID 28006  SPAIN |

## Switzerland

| **Center** | **Institutional Review Board or Ethics Committee Address(es)** |
| --- | --- |
| 1074 * | SPUK fuer Innere Medizin  Prof. Dr. med. Renato Galeazzi  UniversitaetsSpital Zuerich  Sonneggstrasse 12  Zuerich, CH-8091  SWITZERLAND |
| 1076 | Comite d'etique  Departemental de Médecine Interne et Médecine Communautiare  Hôpitaux Universitaires de Genève  4, Rue Gabrielle-Perret Gentil  CH-1211 Genève,  SWITZERLAND |
| 1173 | Commission d'ethique de la recherche clinique  Faculté de Biologie et de Médecine  Décanat  Rue du Bugnon 21  Lausanne, CH-1011  SWITZERLAND |

## Taiwan

| **Center** | **Institutional Review Board or Ethics Committee Address(es)** |
| --- | --- |
| 1160 * | Taipei Veterans General Hospital, The Institutional Review Board  No. 201  Sec. 2, Shih-Pai Road  Taipei, 112  TAIWAN |
| 1161 * | Taichung Veterans General Hospital, The Institutional Review Board  160, Section 3, Taichung Kang Road  Taichung, Taiwan 407  TAIWAN |
| 1162 | Chang Gung Medical Foundation-Linkou Branch  5  Fu-Shin Street  Kweishan, Taoyuan County 333  TAIWAN |
| 1183 | Kaohsiung Medical University Chung-Ho Memorial Hospital  No.100 , Tzyou 1st Road  Kaohsiung, 807  TAIWAN |

## Thailand

| **Center** | **Institutional Review Board or Ethics Committee Address(es)** |
| --- | --- |
| 1155 * | The Khon Kaen University Ethics Committee for Human Research  Faculty of Medicine, Khon Kaen University  Dean Office 6 floor  123 Mitraphap Road  Khon Kaen, 40200  THAILAND |
| 1156 | Chulalongkorn Univ Hosp  Faculty of Medicine  Rama IV Road  Bangkok, 10330  THAILAND |
| 1157 | Institutional Review Board Royal Thai Army Medical Department  Phramongkutklao Hospital, 317  Rachavithee Road  Rachathevee, Bangkok 10400  THAILAND |
| 1158 | Siriraj Ethics Committee  2 Bangkoknoi, Siriraj Hospital, Mahidol University  Prannok Road  Bangkok, 10700  THAILAND |
| 1159 | Ramathibodi Hospital, Mahidol University  270 Faculty of Medicine, Rama IV Road  Bangkok, 10400  THAILAND |

## Turkey

| **Center** | **Institutional Review Board or Ethics Committee Address(es)** |
| --- | --- |
| 1053 | Ege Universitesi Tip Fakultesi Klinik Arastirmalar Etik Kurulu  Ege Universitesi Tip Fakultesi Dekanlik Binasi 2. kat  Izmir/Bornova, Turkiye 35100  TURKEY |
|  | T.R. Ministry of Health General Directorate of Pharmaceuticals and Pharmacy  Sogutozu Mahallesi 2176. Sokak No:5  Cankaya, Ankara 06520  TURKEY |
| 1054 * | Ankara No.3 Clinical Research Ethics Committee  Hacettepe University Medical Faculty  Sihhiye  Ankara, 06100  TURKEY |
|  | T.R. Ministry of Health General Directorate of Pharmaceuticals and Pharmacy  Sogutozu Mahallesi 2176. Sokak No:5  Cankaya, Ankara 06520  TURKEY |
| 1206 | Ege Universitesi Tip Fakultesi Klinik Arastirmalar Etik Kurulu  Ege Universitesi Tip Fakultesi Dekanlik Binasi 2. kat  Izmir/Bornova, Turkiye 35100  TURKEY |
|  | T.R. Ministry of Health General Directorate of Pharmaceuticals and Pharmacy  Sogutozu Mahallesi 2176. Sokak No:5  Cankaya, Ankara 06520  TURKEY |

## United Kingdom

| **Center** | **Institutional Review Board or Ethics Committee Address(es)** |
| --- | --- |
| 1098 * | Northern & Yorkshire MREC  Unit 215  TEDCO Business Centre  Viking Industrial Park  Jarrow, NE32 3DT  UNITED KINGDOM |
| 1099 | Northern and Yorkshire Multi-centre Research Ethics Committee  TEDCO Business Centre  Room 002, Viking Industrial Park  Jarrow  Tyne & Wear, NE32 3DT  UNITED KINGDOM |
|  | South London REC Office (2)  King's College Hospital,Camberwell Building  1st Floor  94 Denmark Hill  London, SE5 9RS  UNITED KINGDOM |
| 1100 * | Northern and Yorkshire Multi-centre Research Ethics Committee  TEDCO Business Centre  Room 002, Viking Industrial Park  Jarrow  Tyne & Wear, NE32 3DT  UNITED KINGDOM |
| 1101 * | Northern and Yorkshire Multi-centre Research Ethics Committee  TEDCO Business Centre  Room 002, Viking Industrial Park  Jarrow  Tyne & Wear, NE32 3DT  UNITED KINGDOM |
| 1193 | Northern and Yorkshire Multi-centre Research Ethics Committee  TEDCO Business Centre  Room 002, Viking Industrial Park  Jarrow  Tyne & Wear, NE32 3DT  UNITED KINGDOM |

## United States

| **Center** | **Institutional Review Board or Ethics Committee Address(es)** |
| --- | --- |
| 1011 * | Western Institutional Review Board, Inc.  3535 Seventh Avenue, SW  Olympia, WA 98502  UNITED STATES |
| 1012 * | Office of Research Protection  1500 East Duarte Road  Duarte, CA 910104  UNITED STATES |
| 1014 | Wake Forest University Health Sciences Institutional Review Board  Medical Center Boulevard  Winston Salem, NC 27157-1042  UNITED STATES |
| 1017 * | Duke University Health System  Institutional Review Board  Suite 405 Hock Plaza  2424 Erwin Road  Durham, NC 27710  UNITED STATES |
| 1018 * | Western Institutional Review Board  3535 Seventh Avenue Southwest  Olympia, WA 98502  UNITED STATES |
| 1020 * | University of Miami  Human Subjects Research Office  Suite 1002  1500 NW 12th Avenue  Miami, FL 33136  UNITED STATES |
| 1021 * | Baylor Research Institute IRB  Baylor University Institutional Review Board  Suite 510  3310 Live Oak Street  Dallas, TX 75204  UNITED STATES |
| 1039 * | University of California Davis Institutional Review Board  Institutional Review Board  CTSC Building  Suite 1400 Room 1429  2921 Stockton Boulevard  Sacramento, CA 95817  UNITED STATES |
| 1040 | Wayne State University  Human Investigation Committee  101 East Alexandrine Building  Detroit, MI 48201  UNITED STATES |
| 1041 | Henry Ford Health System, Institutional Review Board  CFP-Basement 046  2799 West Grand Boulevard  Detroit, MI 48202  UNITED STATES |
| 1051 | Western Institutional Review Board  3535 Seventh Avenue Southwest  Olympia, WA 98502  UNITED STATES |
| 1056 * | HCA HealthONE Institutional Review Board  Suite 265A  720 South Colorado Boulevard  Glendale, CO 80246  UNITED STATES |
| 1065 | University of Chicago IRB  5751 S. Woodlawn Ave.  2nd Floor  Chicago, IL 60637  UNITED STATES |
| 1067 * | Western Institutional Review Board, Inc.  3535 Seventh Avenue, Southwest  Olympia, WA 98508-2029  UNITED STATES |
| 1068 * | Mount Sinai Institutional Review Board  Mount Sinai Medical Center  1 Gustave L. Levy Place  New York, NY 10029  UNITED STATES |
| 1069 * | Northwestern University Institutional Review Board  Rubloff, 7th Floor  750 North Lake Shore Drive  Chicago, IL 60611  UNITED STATES |
| 1078 | Western Institutional Review Board  3535 Seventh Avenue Southwest  Olympia, WA 98502  UNITED STATES |
| 1083 | UAMS Institutional Review Board  University of Arkansas for Medical Services  # 636  4301 W. Markham  Little Rock, AR 72205  UNITED STATES |
| 1086 * | Committee on Investigations Involving Human Subjects for School of Medicine  University of California, San Diego  0052  9500 Gilman Drive  La Jolla, CA 92093-0052  UNITED STATES |
| 1105 * | BRANY IRB  Suite 100  225 Community Drive  Great Neck, NY 11021  UNITED STATES |
| 1106 | University of Pennsylvania  Committee of Studies Involving Human Beings/Office of Regulatory Affairs  Suite 301 South  3624 Market Street  Philadelphia, PA 19104  UNITED STATES |
| 1114 | Fred Hutchinson Cancer Research Center  Institutional Review Office  PO Box 19024  J6-110  Seattle, WA 98109  UNITED STATES |
| 1115 * | Loyola University Institutional Review Board  Building 120, Room 400  2160 South 1st Avenue  Maywood, IL 60153  UNITED STATES |
| 1124 * | Western Institutional Review Board  P.O. Box 12029  3535 Seventh Avenue Southwest  Olympia, WA 98508-2029  UNITED STATES |
| 1140 | Johns Hopkins Medical Institutional Review Board  Reed Hall B130  1620 McElderry Street  Baltimore, MD 21205  UNITED STATES |
| 1141 * | University of Kentucky Medical Institutional Review Board  Office of Research Integrity  315 Kinkead Hall  Lexington, KY 40506-0057  UNITED STATES |
| 1145 * | Human Subject Protection Program  MedCenter One  Suite 200  501 East Broadway  Louisville, KY 40202  UNITED STATES |
| 1150 * | LSU Health Sciences Center IRB  1501 Kings Highway  Shreveport, LA 71103  UNITED STATES |
| 1154 * | Research Subjects' Protection Programs  University of Minnesota  Mayo Mail Code 820  420 Delaware Street  Minneapolis, MN 55455  UNITED STATES |
| 1164 | Oregan Health Sciences University IRB  2525 Southwest 1st Avenue  Portland, OR 97201  UNITED STATES |
| 1165 | Office of Protocol Research  Unit 1009  1515 Holcombe Blvd  Houston, TX 77030  UNITED STATES |
| 1174 * | BRANY IRB  Suite 100  225 Community Drive  Great Neck, NY 11021  UNITED STATES |
| 1179 * | Rush University Medical Center  Institutional Review Board  Research and Clinical Trials Administration Office  1653 W. Congress Parkway  Suite 439 Professional Building  Chicago, IL 60612  UNITED STATES |
| 1188 * | CHRCO IRB  5700 Martin Luther King Jr Way  Oakland, CA 94609  UNITED STATES |
| 1189 | UCSF Committee on Human Research, Office of Research  Suite 315  3333 California Street/ Box 0692  San Francisco, CA 94118  UNITED STATES |
| 1190 * | Western Institutional Review Board  3535 Seventh Avenue Southwest  Olympia, WA 98502  UNITED STATES |
| 1191 | Office for the Protection of Research Subjects  203 Administrative Office Building  1737 West Polk Street  Chicago, IL 60612  UNITED STATES |
| 1194 * | West Virginia University Institutional Review Board  886 Chestnut Ridge Road  Morgantown, WV 26506  UNITED STATES |
| 1195 * | Institutional Review Board  Office of Regulatory Affairs  987830 Nebraska Medical Center  Omaha, NE 69198-7830  UNITED STATES |
| 1198 * | Tufts Medical Center Institutional Review Board  Box 817  800 Washington Street  Boston, MA 02111  UNITED STATES |
| 1201 | Western Institutional Review Board  3535 Seventh Avenue Southwest  Olympia, WA 98502  UNITED STATES |
| 1203 | Western Institutional Review Board  3535 Seventh Avenue Southwest  Olympia, WA 98502  UNITED STATES |
| 1205 * | Biomedical IRB  University of North Carolina  Medical School Building #52 CB #7097  Chapel Hill, NC 27599  UNITED STATES |
| 1207 * | Fox Commercial  Institutional Review Board  Suite 218  326 North Seventh Street  Springfield, IL 62701  UNITED STATES |
| 1208 * | Roper St. Francis Hospital IRB  316 Calhoun Street  Charleston, SC 29401  UNITED STATES |
| 1210 * | University of Kansas Medical Center  Human Subjects Committee  3901 Rainbow Boulevard  Kansas City, KS 66160  UNITED STATES |
| 1230 * | Chesapeake Research Review, Incorporated  Suite 110  7063 Columbia Gateway Drive  Columbia, MD 21046  UNITED STATES |
